# The SMARTIE Program: A Novel Initiative to Evaluate the Impact of AP Continuing Education on Clinical Practice

**Published:** 2017-11-01

**Authors:** Sandra E. Kurtin, Constance Visovsky, Erik D. Brady, Alana L. K. Brody

**Affiliations:** 1 University of Arizona Cancer Center, Tucson, Arizona;; 2 University of South Florida College of Nursing, Tampa, Florida;; 3 EDBPhD Consulting, LLC, Winston-Salem, North Carolina;; 4 Harborside Medical Education, Lawrenceville, New Jersey

The landscape of cancer care in the United States continues to undergo numerous shifts and innovations, with notable progress in drug development and technologies, resulting in improved progression-free and overall survival for many cancer diagnoses (American Society of Clinical Oncology [ASCO], 2016). Nevertheless, the nation’s cancer care delivery system remains rife with substantial challenges, including access to care, unsustainable rising costs of care, and an aging population that is both expanding in size and living longer with cancer, with many who will require ongoing monitoring and treatment. Between 2010 and 2030, a 45% increase in cancer incidence is expected, which will further increase the demand for cancer care and services (Smith, Smith, Hurria, Hortobagyi, & Buchholz, 2009). However, a steadily growing proportion (19.8%) of oncologists is nearing retirement age (ASCO, 2015), and older oncologists continue to outnumber the 13.9% of oncologists less than 40 years old who have recently entered the field (ASCO, 2016). Results of a study commissioned by ASCO in 2007 indicate that the demand for oncology visits is expected to increase 48% by 2020, while the supply of oncologists will increase by only 14% (Towle et al., 2011). 

With the number of practicing oncologists expected to decline in the coming decades, advanced practitioners (APs) in oncology will become increasingly vital in delivering care and services to the ever-expanding population of cancer patients (Reynolds & McCoy, 2016). Oncology APs, who are fundamental to the cancer care workforce and fulfill a variety of roles across practice settings and states, include nurse practitioners (NPs), physician assistants (PAs), clinical pharmacists (PharmDs), clinical nurse specialists, and other nurses with advanced degrees (Kurtin et al., 2015). All oncology APs are trained at the master’s level or above and manage myriad aspects of care that include cancer diagnostics and related procedures, cancer treatments, symptom management, medication prescribing and management, evaluation of treatment response, survivorship care, and palliative/supportive care (Kurtin et al., 2015). According to the 2016 ASCO Census, 73% of practices employ APs, an increase from the 52% noted in the 2014 Census (ASCO, 2016). Oncologists working collaboratively with APs report improved efficiency, higher professional satisfaction, and improved patient satisfaction (Towle et al., 2011). 

The frenetic pace of clinical development and scientific innovation in oncology has resulted in numerous changes to standards of care across cancer diagnostic groups. Such changes are potentially outpacing the ability of oncology care providers to assimilate and use new information in a timely way (ASCO, 2017). Effective integration of changing standards of care into clinical practice demands innovative strategies for continuing education (CE) and necessitates careful measurement of outcomes. A need exists to meet the CE requirements of oncology APs in a manner that is efficient and cost-effective, while promoting teamwork and professional development. Consistent with the concept of integrated care currently pervasive in the oncology community, APs need to be informed about collaborative practice and interprofessional care to enhance their role and contributions, regardless of experience level. Learning needs among oncology APs may vary according to level of experience, the practice setting, and the makeup of the interdisciplinary team. Whereas APs who are newer to oncology need resources to assist with their orientation to an oncology practice, APs who are more experienced need resources to support the lifelong learning required by the continuous changes in oncology practice. Therefore, CE for oncology APs requires a broad focus that allows individual APs to tailor their education based on their experience and practice environment. 

In the current CE climate, outcomes measurement plays an increasingly important role. The CE community continues to seek new models and methods to quantify the ability of learners to translate new knowledge into practice. Moore and colleagues’ outcomes model has served as a reliable template for measuring educational outcomes in CE in response to the delivered education (Moore, Green, & Gallis, 2009). But the Moore model, while providing an adequate framework for measuring change in individual learners, does not effectively assess the learner’s ability to effect change within the system or practice. The Expanded Learning Model for Systems (TELMS), which includes a series of four behavior and learning stages, is meant to address this gap by complementing Moore’s model within a system-based framework. Compared with Moore’s individual learner–focused framework, the TELMS model is designed to measure the ability of the individual to incorporate learning into practice *within his or her specific health system* (Ruggiero, Robinson, & Paynter, 2015). As such, the TELMS model is best considered a useful extension of Moore’s model, rather than a replacement for it. 

Established in 2014, the Advanced Practitioner Society for Hematology and Oncology (APSHO) is a relatively new society for APs in oncology. The core of the APSHO mission is fostering professional development and facilitating collaborative practice across the cancer care continuum in a variety of practice settings. Thus, APSHO aims to improve the quality of care for patients with cancer largely through professional education. The Study to Measure Advanced Practitioner Retention of Targeted Information and Education (SMARTIE) was initiated to provide quantitative feedback about the quality of education at the JADPRO Live at APSHO 2016 conference in National Harbor, Maryland. Using a conference-based approach, SMARTIE represents a novel initiative for measuring learning and how it is applied to clinical practice by oncology APs.

## STATEMENT OF NEED

In a recent survey conducted by the American Hospital Association that asked members to rate the value of continuing medical education (CME) and its overall effectiveness in addressing core competencies, many members felt that existing CME failed to emphasize the importance of clinical integration, performance improvement, and system-based practice (Combes & Arespacochaga, 2014). The commitment to practice change (CTC) has been used as an evaluation tool for health-care educational programs to document self-reported practice changes, examine the impact of an educational activity, and improve understanding of the learning-to-change continuum in a given clinical area (Shershneva, Wang, Lindeman, Savoy, & Olson, 2010). With healthcare delivery moving toward an interprofessional team-based approach, there is also a greater need for CE initiatives to embrace team-based and interprofessional training. 

Oncology APs are required to adopt evidence-based clinical behaviors and practices that improve patient outcomes. Interprofessional collaborative practice models are among the most common practice models reported by APs in oncology. Therefore, educational interventions or learning systems that facilitate the effectiveness of the interprofessional health-care team in improving patient and caregiver outcomes through knowledge transfer and enhanced communication should be the goal.

**Figure 1:**
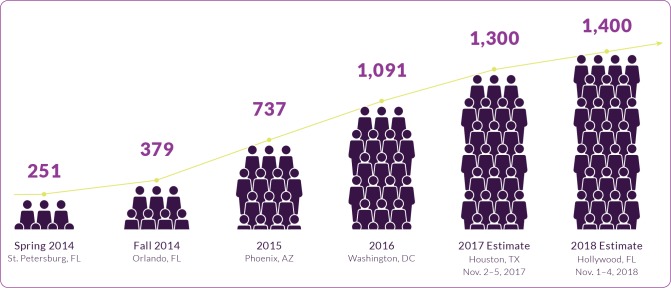
Advanced practitioners are attending JADPRO Live at APSHO in increasing numbers.

The JADPRO Live at APSHO meeting, initially held in January 2014, is an annual conference that focuses on the specific educational needs of APs in oncology and seeks to assist APs in increasing their oncology knowledge base and transferring that knowledge to improve patient care. Since 2014, attendance at JADPRO Live at APSHO has steadily increased and has attracted a diverse range of APs, including pharmacists, nurse practitioners, and PAs as both attendees and members of APSHO (Figure 1). 

## SMARTIE FRAMEWORK

The SMARTIE program was initiated based on the need to determine if APs are receiving CE knowledge in a manner that is effective and meaningful; the application of knowledge to clinical practice; and the impact of the CE on provider knowledge, practices, and patient outcomes. Instituting this programmatic approach and incentivizing attendees to participate in the measurement helped obtain the metrics for the data reported. The framework for the JADPRO Live at APSHO conference incorporated the methods of Moore’s model by using multiple-choice questions to assess competence or knowledge change. 

The inaugural SMARTIE initiative included a total of 14 topics specific to oncology: chemotherapy-induced nausea and vomiting (CINV), head and neck cancers, gastric cancer, skin cancer, chimeric antigen receptor T-cell (CAR-T) therapy, aggressive lymphomas, chronic lymphocytic leukemia (CLL), non–small cell lung cancer (NSCLC), venous thromboembolism (VTE), immunotherapy, hormone receptor–positive breast cancer, multiple myeloma, and pancreatic cancer. 

## METHODS

**Figure 2:**
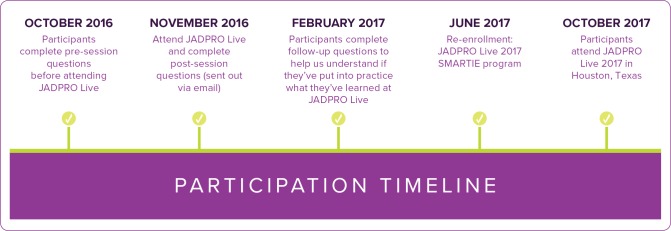
Timeline depicting key milestones in the development of the SMARTIE initiative.

For a timeline showing key milestones in the development of the SMARTIE program, see Figure 2.

**Recruitment**

The target for recruitment for the SMARTIE program was set at 200 JADPRO Live attendees. A total of 207 participants completed the pre-conference questions.

Prospective participants were recruited via a series of email communications sent to members of APSHO describing the SMARTIE program. Interested members proceeded to a link for registration, which included basic demographic information and a signed agreement for participation. Members felt to be engaged in organization activities, including APSHO committee members, JADPRO Editorial Board members, and those who had attended at least two JADPRO Live conferences, were targeted in the first round of emails. If those individuals had not registered after at least four emails, they received a follow-up phone call from the JADPRO Editorial Director. The second phase of recruitment focused on all remaining APSHO members and those who had attended any past JADPRO Live conference. A digital ad linked to the SMARTIE sign-up webpage was added to the JADPRO Live conference registration webpage to open up participation to interested registrants. 

Several incentives were offered to those who agreed to participate in the SMARTIE program:

- A 75% discounted tuition for the 2016 conference
- Free JADPRO Live 2017 conference tuition with 100% completion of all 2016 SMARTIE requirements
- Direct input into priorities for educational content at future JADPRO Live meetings.

**Requirements and Measures**

The 14 oncology topics mentioned previously were identified by the conference planning committee as SMARTIE educational sessions. In order to provide evaluative data, speakers assigned to those sessions were asked to create pre- and post-test questions for their session. Speakers were provided instruction and guidance on question-writing techniques (Brady, 2015). The pre- and post-test questions were designed to measure knowledge before and after participation in the SMARTIE educational sessions. Each question was structured to have five answer options, including an "unsure" option, which was incorporated to (1) reduce the probability of a guess of the correct answer, leading to an inaccurate measure of knowledge gain; and (2) provide an indication of ongoing learner need to help inform future JADPRO Live conferences. Documenting the percentage of learners who deliberately select "unsure" as the answer choice at baseline provides insight into knowledge gaps and may be indicative of educational need for targeted learners beyond those attending the conference. 

The new SMARTIE initiative collected participants’ responses to pre/post-session questions online, in contrast to the JADPRO Live at APSHO 2015 conference, during which evaluation questions were presented on slides at the beginning and end of each educational session, utilizing an audience response system. While this change in methodology may seem counterintuitive with respect to maximizing engagement in the outcomes study, this method was highly effective with APs attending JADPRO Live.

To satisfy the SMARTIE program requirements, participants were required to:

- Complete 50 pre-session knowledge or competence assessment questions covering the 14 SMARTIE-designated conference sessions prior to the start of the conference. The links to pre-session questions were sent to participants via email, with an option to complete all questions onsite upon arrival at the conference
- Attend 9 of the 14 SMARTIE-designated sessions and complete the post-session knowledge or competence assessment questions for those sessions.

**Timeline**

Three weeks prior to the conference, an email with a link to pre-session questions was sent to all participants, with reminders sent to those with incomplete answers. Upon arrival at the conference, SMARTIE enrollees received a postcard with SMARTIE information/instructions in their welcome bag. Participants with incomplete pre-tests were not permitted to receive their conference badge until the pre-test was completed. 

During the conference a SMARTIE Station, consisting of a booth monitored by APSHO staff, was located outside the main conference ballroom. APSHO staff were equipped with iPads to allow participants to complete their SMARTIE-related questions, or questions could be answered on personal laptop or mobile devices. In addition, participants received a link to post-session questions via email twice daily on the Friday and Saturday of the conference and once on Sunday, as the conference ended. After the conference, three emails with a link to post-test questions were sent to participants who had not yet completed them. Participants were allowed up to 1 month after the conference ended to complete post-test questions. 

**Figure 3:**
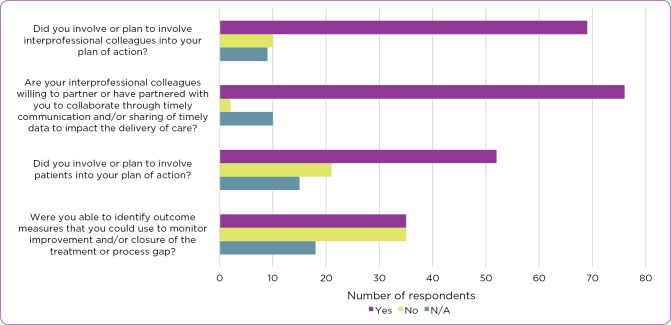
Strategies deployed by learners at 16-week follow-up.

About 4 months after the conference, three emails with a link to long-term follow-up questions were sent to participants requesting information highlighting aspects of learning incorporated into clinical practice. Whereas the live conference component of the SMARTIE program used multiple-choice questions to gauge learning, the follow-up component of the program incorporated questions based on rating scales or open-ended responses, intended to more accurately quantify change within participants’ health system and practice area. Additionally, a survey, developed to assess system-based outcomes consistent with the TELMS model, was part of the follow-up component. As part of the follow-up survey, participants answered a series of questions related to strategies deployed in applying knowledge from the SMARTIE sessions to everyday practice (Figure 3). These strategies were aligned with National Quality Strategy (NQS) priorities, which pursue the broad aims of better care, healthy people/healthy communities, and affordable care to guide and assess efforts to improve health and healthcare quality (Agency for Healthcare Research and Quality [AHRQ], 2016). 

## RESULTS

**Figure 4:**
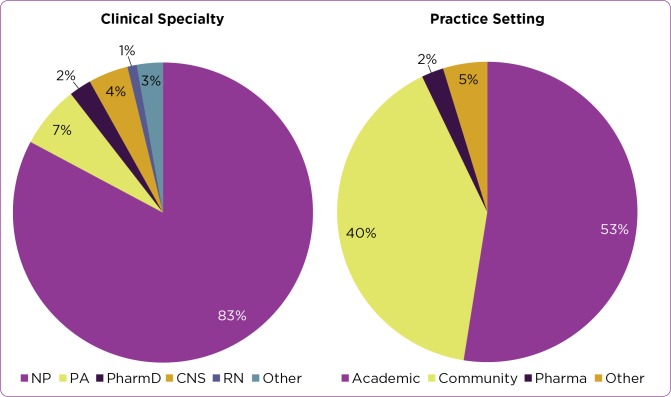
Profile of SMARTIE participants according to clinical specialty and practice setting.

Because individuals enrolled in SMARTIE were intended to be representative of the overall learner population, there were no substantial differences between the demographics of those enrolled in this program and the overall population of attendees at the 2016 conference. SMARTIE enrollees represented various clinical disciplines and practice settings, with the majority being NPs from both academic and community practice settings (Figure 4). Additionally, 70% of NPs practice independently with physician oversight and 81% of NPs report the ability to prescribe independently.

The designers of the study measured session engagement by the number of people who answered post-test questions; that is, the more questions answered by participants after a session, the more engaged participants were with that session. At the 2015 meeting, the educational session associated with the highest level of engagement had only 30 people answering post-test questions. In contrast, the session associated with the highest level of engagement at the 2016 conference had 183 people answering post-test questions, a six-fold increase. See Table 1 for more results, including the participation numbers for the session with the least-engaged audience and those for a session with a moderately engaged audience, broken out by year.

**Table 1:**
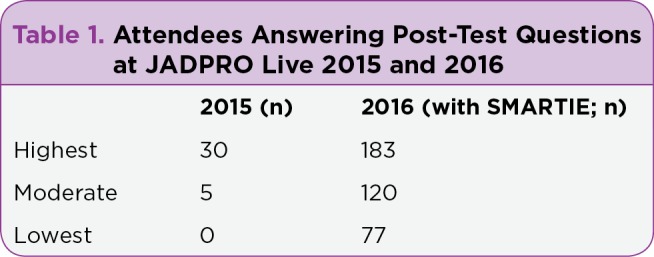
Attendees Answering Post-Test Questions at JADPRO Live 2015 and 2016

**Figure 5:**
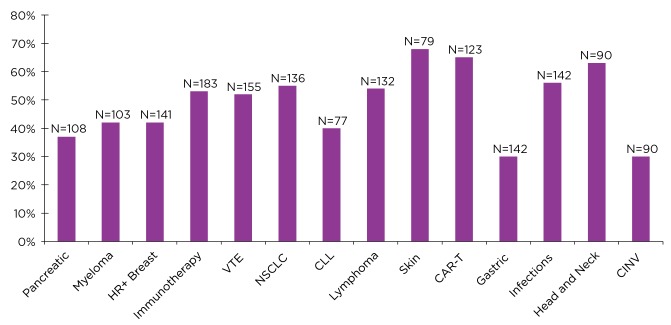
Increase in knowledge of participants at 14 designated SMARTIE sessions at the 2016 JADPRO Live at APSHO meeting. HR+ = hormone receptor–positive; VTE = venous thromboembolism; NSCLC = non–small cell lung cancer; CLL = chronic lymphocytic leukemia; CAR-T = chimeric antigen receptor T-cell; CINV = chemotherapy-induced nausea and vomiting.

At the 2016 conference, 88% of SMARTIE learners participated in both the pre- and post-tests. This level of engagement extended into the knowledge and competence transfer as well, as evidenced by a 39% increase in knowledge among participants in the 14 SMARTIE sessions across the conference. The areas of skin cancer, CAR-T, and head and neck cancer had the highest increases in knowledge attainment (Figure 5). 

An example of the effectiveness of the SMARTIE program is demonstrated by the following data collected at the conference. For SMARTIE participants attending the session on CINV, learners showed significant (*p* < .0001) improvement in their ability to recall chemotherapy agents that are highly and moderately emetogenic, with a 30% increase in knowledge gained from pre- to post-test. The ability of APs to anticipate and prevent CINV increased 18% from pre- to post-test. SMARTIE participants also demonstrated significant improvements in knowledge from baseline to post-test in multiple areas of oncology treatment (Table 2). 

**Table 2:**
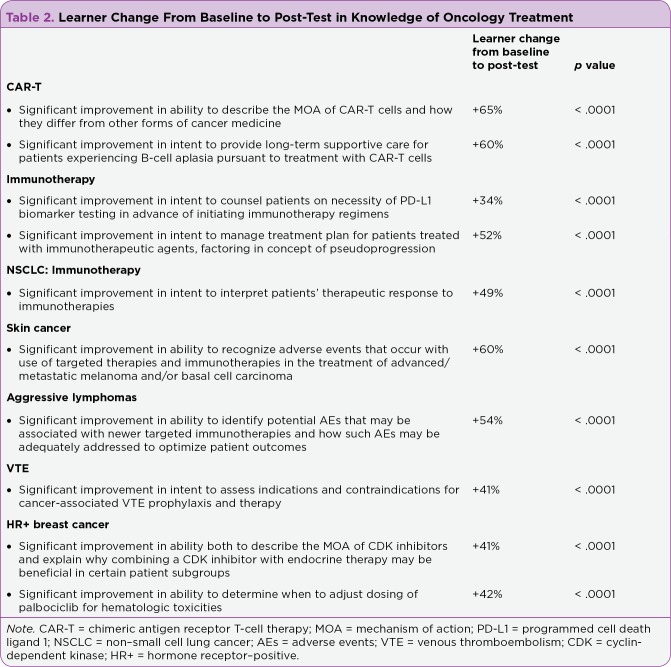
Learner Change From Baseline to Post-Test in Knowledge of Oncology Treatment

At the 16-week post-conference follow-up, 88 of 182 SMARTIE participants (48%) responded to the follow-up survey. Approximately 3,000 patients were reported as positively impacted by the 88 respondents, and extrapolation to the entire learner base of JADPRO Live suggests that approximately 35,000 patients may have been positively impacted by this education. 

Figure 3 illustrates the extent to which learning strategies were deployed by SMARTIE participants. In reference to specific learner strategies deployed, 78% indicated an increased awareness within their teams of needed change (the "activate" stage of TELMS); 86% expressed that their health-care teams have collaborated to convert information gained from the conference into planned action (the "advance" stage of TELMS); and 40% acknowledged that their teams had been able to demonstrate engagement by identifying specific measures for monitoring an identified gap in health care (the "aspire" stage of TELMS).

**Figure 6:**
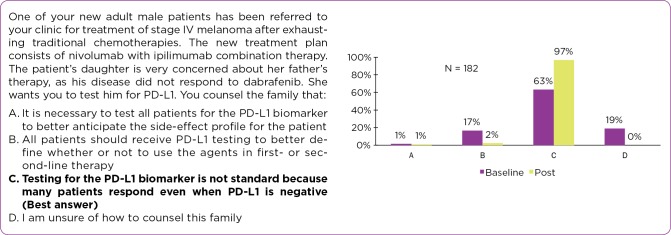
Outcomes of intention to counsel patients on the necessity of PD-L1 biomarker testing.

Among the valuable insights that emerged from the follow-up survey, learners showed a significant improvement in their ability to counsel patients on the appropriateness of PD-L1 biomarker testing in advance of initiating immunotherapy regimens (Figure 6). Whereas participants were not aware of the appropriate situations for counseling their patients on PD-L1 biomarker testing prior to the conference, they were counseling patients about this relevant aspect of immunotherapy after the conference, as a result of knowledge acquired. 

As part of the follow-up study, selected anecdotal self-reported outcomes by learners were specified according to the identified gap in practice, the strategy or proposed practice change to address the gap, and the plan for monitoring the treatment/process gap in the individual’s practice. A summary of the identified practice gaps is shown in Table 3. More in-depth analysis of the SMARTIE program is planned, with the intention to examine longer-term outcomes from participants who attend JADPRO Live at APSHO again in 2017.

**Table 3:**
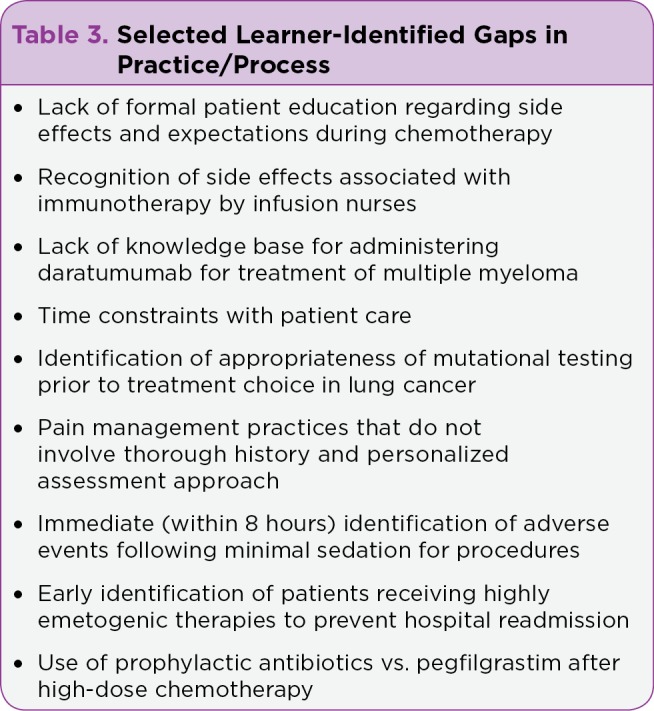
Selected Learner-Identified Gaps in Practice/Process

An example of the application of knowledge to the practice setting—a fundamental concept of SMARTIE—pertained to the identification of immunotherapy-related adverse events (Table 4). One outcome related to immunotherapy revealed that a full 91% of learners trained their *team members* about immunotherapy-related adverse events (59% trained 1 to 9 members; 7% trained 10 to 20 members; 25% trained > 20 members). 

**Table 4:**
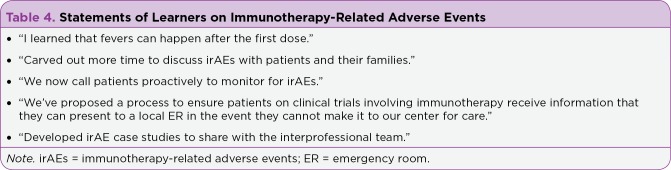
Statements of Learners on Immunotherapy-Related Adverse Events

With respect to alignment with NQS, learners identified four of six NQS priorities as clearly aligned with the JADPRO Live at APSHO conference:

- Promoting effective communication/coordination of care (85%)
- Promoting effective prevention and treatment practices (77%)
- Ensuring patient and family engagement in care (67%)
- Making care safer by reducing harm in care delivery (64%).

The two remaining NQS priorities, judged to be less evident but not absent, were making care more affordable using new models (36%) and working in communities to enable healthy living (18%). 

## DISCUSSION

With the landscape of cancer care and treatment rapidly changing, there is an ongoing need for oncology APs to obtain high-quality CE that is delivered in a manner that is both efficient and cost-effective, with content and format that foster collaborative practice. Although there are certain limitations associated with traditional methods for measuring CE outcomes, and more refined methods are expanding our understanding of the true extent to which APs integrate CE knowledge into practice in a meaningful way, improvements have been made in practice following participation in JADPRO Live. At best, these methods are a proxy for change, but they do provide an indication of how health-care providers intend to change, which is a critical precursor to performance change in the clinician’s practice setting. 

Quantitative feedback from participants is optimal in order to improve education and continue to provide quality CE. Based on feedback from CE activity organizers, past efforts related to obtaining quantitative feedback from JADPRO Live were less successful owing to suboptimal engagement of the conference attendees in outcome evaluations. With SMARTIE, we sought to create a more unique learning initiative that was focused on engagement in the outcomes study by offering incentives to limit attrition. To date, the SMARTIE program has yielded some powerful data that will drive more effective outcomes measurement and help identify trends in how learning is effectively and efficiently applied by APs in their practice settings. 

JADPRO Live may be differentiated from other CE programs for APs in its focus on and commitment to interprofessional collaboration. As a professional educational activity, JADPRO Live at APSHO is focused on four main actions:

- Interprofessional teamwork and team-based practice
- Interprofessional communication practices
- Values/ethics for interprofessional practice
- Roles and responsibilities for collaborative practice.

SMARTIE enrollees were particularly dedicated to participating in all aspects of the outcomes model. The 48% completion rate for the SMARTIE follow-up survey is worth noting, as this exceeds typical response rates to surveys (Cook et al., 2010). In contrast to other CE programs for APs, SMARTIE demonstrated a robust response 4 months after the JADPRO Live meeting. In addition, many of the learners shared detailed anecdotes on how they are implementing their learning into practice. These responses show specific alignment with the NQS Priorities as defined by the AHRQ (2016). 

Deployment of learner strategies demonstrated that a percentage of learners involved both interprofessional colleagues and patients in their plan of action. Without the support of the interprofessional health-care team, an individual learner may face challenges in trying to implement new strategies for treatment and patient care. In some cases, learners had already had an opportunity to practice what they learned through SMARTIE sessions; in other cases, learners had not had an opportunity to practice what they learned because they did not see a certain type of cancer patient. 

Several strengths are associated with the SMARTIE program. To begin, the conference-based approach of SMARTIE allowed for a variety of oncology APs to be reached and included learning strategies that were well developed and measurable. It is often difficult to engage participation in educational initiatives, and the SMARTIE program was unique in that it focused on active participation by APs. The program was designed to meet specific learning needs identified by stakeholders, which were defined from a prior JADPRO Live conference survey. Evaluations of SMARTIE were conducted separately from those of the overall conference evaluations, which allowed learner outcomes to be tied to the actual participants. Anecdotal self-reported outcomes by SMARTIE participants helped support the quantitative data. Specific CE needs were identified in the 16-week follow-up component of SMARTIE. 

While there is a risk associated with measuring outcomes at conference events, we have found that oncology APs are a group that is willing to fully engage with the SMARTIE outcomes model. However, even with incentives, not all SMARTIE participants fulfilled the required commitment. The extent to which "unsure" as a response choice provided useful data for program improvement is not clear but may be an effective measure of learning. The lack of beginner or basic oncology sessions in SMARTIE to address the needs of newer practitioners with limited oncology experience is a recognized limitation of this initiative. Despite the growing attendance at JADPRO Live, the completion of post-tests to measure learning remained low at 32%, limiting the ability to effectively measure learning and adapt future educational initiatives. 

## CONCLUSIONS

Overall, the SMARTIE program was executed successfully, with self-reported implementation of learned concepts in the oncology clinical setting, consistent with Level 5 of Moore’s educational model. SMARTIE participants agree that the information learned in the required sessions was beneficial in helping them treat their oncology patients. Moreover, results from the immediate and follow-up portions of the SMARTIE program showed that participants gained crucial knowledge in relevant areas of oncology (e.g., immunotherapy) and implemented that knowledge in their clinical practice settings. 

With respect to future recommendations, data gathered thus far will be further analyzed by the SMARTIE planning committee and considered in the development of future educational activities.

**Acknowledgments**

The authors would like to thank Lisa van Devender, PharmD, for her expertise and assistance in preparing this white paper. Please see the Appendix for a list of learners whose efforts made this initiative possible. 

## Appendix

**Thank you to our SMARTIE Taskforce**

Sandra E. Kurtin, PhDc, ANP-C, AOCN®

University of Arizona Cancer Center

Pamela H. Viale, RN, MS, CNS, ANP

Advanced Practitioner Society for Hematology and Oncology

Constance Visovsky, PhD, RN, ACNP, FAAN

University of South Florida

Carolyn M. Grande, CRNP, AOCNP®

University of Pennsylvania

Erik D. Brady, PhD, CHCP

EDBPhD Consulting, LLC

Alana L. K. Brody, MBA, CHCP

Harborside Medical Education

**Thank you to our JADPRO Live at APSHO 2016 SMARTIE participants**

Deborah Abu-Alrub

Nancee Albright

Terrinda Alston

Catherine Anderson

Lisa Arcikowski

Jennifer Armendariz

Angela Bazzell

Ana Begley

Catherine Bishop

Myra Blankenship

Hannah Blondke

Chelsey Boggs

Sarah Bonerigo

Amy Bradbery

Danielle Brandy

Kevin Brigle

Laurie Bryant

Gail Bujorian

Katherine Bukolt

Kelly Burtch

Jennifer Byrne

Michelle Camarda

Thanh Cao

Rachael Carter

Cheryl Casella

Peggy Cave

Amos Chery

Leigh Childress

Joan Clark

Joan Collett

Wendy Crabbe

Kelly Curran

Jami Cynecki

Hillary Daver

Marianne Davies

Candace DeBerardinis

Carina DeCroes

Asha Demla

Emily Dill

James Drechsler

Gloria Drouin

Adrienne Duckworth

Rosalie El-Rady

Sheryl Etnier

Sharon Etris

Phyllis Everett

Laurie Farrell

Shaunta Ford-Pierce

Amanda Fowler

Nataya Francis

Jan Garza-Dennis

Nancy Gerum

Alice Gilhool

Paige Goforth

Poonam Goswami

Samantha Greene

Carol Guarnieri

Mistie Hagaman

Susan Hamlin

Maria Hanik

Megan Harrington

Tina Harris

Kara Hart

Lois Hathorn

Karen Henry

Kimberly Hicks

Teresa Hill

Amy Hillsman

Kirsten Hinson

Nancee Hirano

Cindy Jo Horrell

Helena Iannaccone

Antonita Itchon

Jane Johansson

Diane Jones

Michelle Karlin

Patricia Karwan

Elizabeth Keating

Jacquie Kelley

Dawn Key

Elisabeth King

Donna Kinzler

Ruta Kirstein

Patty Kormanik

Kristen Kreamer

Louise Kressly

Tracy Krimmel

Sandra Kurtin

Bernadette Labriola

Ami Lam

Mary Jane LaRoche

Heidi LaRue

Korrina Lau

Kathy Leonard

Heather Lewin

Jennifer Lindsey

Christine Long

Alicia Lowery

Lindsey Lyle

Lynn Magrum

Anne Markham

Kathleen Martin

Brenda Martone

Megan May

Kelley Mayden

Melanie Maze

Rebecca McAlpin

Suzanne McGettigan

Jonlyn McGettigan

Pamela McLean

Nancy Menning

Marcia Mickle

Gena Middleton

Felisha Miller

Mary Lynn Miners

Debra Morris

Theresa Mortensen

Cynthia Murray

Ellen Nason

Fran Necci

Anne Neugent

Kim Nichols

Nancy Nix

Amanda Norton

Julie Nugent

Siobhan O’Donnell

Angela Orrico

Cecilia Otoo

Amy Ottman

Gwendolyn Parker

Cathy Payne

Dawn Paynter

Michele Peetz

Rasheda Persinger

Rebecca Phillips

Victoria Poillucci

Jessica Portu

Victoria Pressling

Alicia Price

Allyson Price

Jaime Rees

Kasandra Risch

Karen Roesser

Barbara Rogers

Robin Rome

Kelley Rone

Alison Ryan

Kathleen Saas

Beth Sandy

Linda Schaefer

Cheryl Schimers

Tyra Schlabach

Kim Schmidt

Cindy Scott

Julie Scott

Anne Marie Shaftic

Kathy Sharp

Brenda Shelton

Gary Shelton

Victoria Sherry

Cyndy Simonson

Marybeth Singer

Debbie Solom

Mikee Spaulding

Julie Spears

Rochele Spires

Aimee Strong

Kirsten Stuber

Robin Suess

Marsha Sundman

Lisa Sweeney

Colleen Sweetland

Geline Joy Tamayo

Siok-Hian Tay-Kelley

Elizabeth Taylor

Sara Toth

Karley Trautman

Rebekah Vega

Jamee Vester

Amanda Viereck

Wendy Vogel

Sandra Voss

Connie Wagenknecht

Kelly Ward

Ashley Webber

Monica White

Megan Wholey

Judith Williamson

Stephanie Wilson

Susan Wilson

Kathleen Witkowski

Susan Woodward

Renee Yanke

Kelly Young

Nelli Zafman

Gillian Zeile

Laura Zitella

Anne Zobec
